# The Union of Dental and Medical School

**Published:** 1900-06

**Authors:** C. S. Minot

**Affiliations:** Boston, Mass.


### THE UNION OF DENTAL AND MEDICAL SCHOOL.[1]

BY DR. C. S. MINOT, BOSTON, MASS.[2]

Mr. President and Gentlemen,—Being a Bostonian, I am always coming to the Common, and I am struck with the sandwich man. These gentlemen, as you know, have going before them and coming behind them parts in which the public is supposed to be interested, and which are worthy of your attention. The man himself is worthy of no consideration whatever, and I feel to-night as a sort of a sandwich man. Those who came before me you have attended to, and those who come after me you should attend to. I am the sandwich man.

One speaker to-night seems to have omitted something I expected him to say,—that is, my honorable friend, the Speaker of the House, Mr. Myers, who privately informed me that he regarded the Democratic party as a case of caries on the tooth of time. I have been fortunate in my situation in one respect, in that I receive confidences. I am merely the sandwich or middle man. My neighbor on my right has informed me that he is the end man, and that he will make all the good jokes of the evening. You need not, therefore, expect me to be anything but serious and uninteresting.

---

[1] Read before the union meeting of The New York Institute of Stomatology and the American Academy of Dental Science, Boston, March 21, 1900.

[2] Professor in Harvard Medical School.

I am very glad personally that President Eliot has found it necessary to withdraw from our meeting, because that gives me more freedom than I would otherwise have felt in speaking of his contribution to the welfare of the dental profession. It seems to me there is no man to whom the dental profession is in greater debt than to President Eliot. It was to his initiative that was due the foundation of the Harvard Dental School, and he has followed up his interest in it, and he alone is responsible for the first suggestion of the change which leads to the topic upon which I have the honor to speak, the consolidation of, and the establishment of intimate relations between, our Dental Schools and our Medical Schools.

I cannot go along any of our streets in Boston without seeing here and there those curious square posts wound around with stripes of black and white. They recall the history of the application of mechanical treatment to human suffering. Every barber's pole is a record held up, for every passer-by to see, that surgery, one of the most important and dignified branches of medicine, started in a most humble fashion. It was a mere collateral occupation of the barber, and from that humble, unpromising beginning the surgical work of the profession has risen to the wonderful dignity of to-day. These facts must give much encouragement to the members of the dental profession. The dentists, like the surgeons, started as people who applied a mechanical treatment to the human body, and I believe that as the surgeon has risen to a recognized position as a person of the highest attainments, worthy to rank side by side with the men of the greatest learning of any profession, so too the dentist should rise from the beginning as a mechanical practitioner to one who is completely a member of the medical profession. I think hardly too much can be urged in this way, because the gain which stands before us is so very great. If we can bring up the dental profession to that point in which it shall take its legitimate position, we shall have done a very great service indeed. Dentistry, viewed in its proper light, is not something which is mere mechanics. Every dentist here, I believe, shares with me the conviction that dentistry should be something more scientific, more learned, than the mere mechanical treatment of caries of the teeth. It is a profession which requires a great deal of scientific knowledge. I hold it to be no criticism of the dental profession that those of us who stand to-day as the teachers were inadequately trained.

We did not have the opportunities, the knowledge did not exist, teachers could not, therefore, give it to us. We live in an age in which knowledge has been constantly added to, and it is a part of our duty, as it seems to me, to add to the range and fulness of the instruction which shall lead to the development of the accomplished dentist of the future. No profession which stands upon its present basis, and which does not go beyond that basis, can ever hope to rise beyond a dead level of monotonous and deathlike repetition of what has been before. We must do something better. As a naturalist I think as a naturalist, therefore I think always that evolution, progress, the going on to something higher and better than that which has ever been, is the very condition of existence, and I believe that the medical profession has gained its high position in society and that it has gone ahead for this reason, and I believe the dental profession is gaining more and more, day by day, because it too is developing a broader stand-point; it too is learning to take the scientific view of the responsibilities of each individual practitioner. The dentist can no longer be a mere mechanic. It is, I believe, the part of those who are engaged with the dental schools to contribute to the advancement of dental education.

It seems to me that perhaps the greatest of the services which President Eliot has rendered the dental school is the bringing about the union, as it is now accomplished at Harvard, of the Dental, the Medical, and the Veterinary Schools. One of the first results has been to raise the standard of entrance to the Harvard Dental School. The next great change to be required is chemistry as a condition of entrance to the school. Certainly that can have but one result,—the ultimate raising of the standard of that school until is as high as in the highest medical school. Then, I think, will come about the amalgamation of these two things. I am convinced that the dental profession will soon be merged absolutely, without demarcation, into the medical profession, perhaps not in one individual's lifetime, but in a short time as measured in the history of civilization and the development of a nation. That means, perhaps, not in ten years or fifteen years, but in some longer period. If we can accomplish it in ten or fifteen years, so much the better for all concerned. When this result comes about, it seems to me there is but one way in which the dental profession can take its proper place in relation to medicine. We know now there are ophthalmology, obstetrics, and gynæcology, and so on through many subjects, making altogether a long list of specialties in medicine. The very growth of medicine contributes to the recognition and importance of specialties. It becomes us now to offer to the students studies which shall be adapted to the special trend of their intellects. Students can now enter some of our medical schools, and if they feel an inclination to special subjects, pursue those alone. They can turn to ophthalmology, obstetrics, or gynæcology, as the case may be. Why not then recognize that the general medical training which gives a knowledge of the general principles of the maintenance of health is as applicable to the practice of dentistry as to that of ophthalmology or obstetrics or gynæcology. Would not that change, the full recognition of that stand-point, place the dental profession in its proper position in regard to the medical profession? It seems to me the change which President Eliot has brought to a fulfilment at Harvard must tend to that one ultimate condition, the incorporation of the dental school in the medical school. Dentistry would then be a specialty in medicine, and such it should become, and I believe that that change in the relation of the dental to the medical profession would redound to the interests of both. I could enlarge very much on this subject, because it is one that has occupied my attention. I would like to add, that when the proposition to unite the two schools was brought forward by President Eliot it seemed to me unwise and unpractical, but a more thoughtful examination of the reasons advanced by him in favor of such a consolidation convinced me that his position was reasonable, and I believe that President Eliot in advocating, and through his personal influence accomplishing, the actual consolidation of the Dental and Medical Schools of Harvard University has made a very great contribution to the welfare of the medical profession, and a contribution, it seems to me, equally great to the welfare of the dental profession. I hold that the benefit is mutual, that the union of these two schools will help each of them, and for that reason I believe that we should all recognize with gratitude the services which President Eliot has rendered to the Dental and the Medical Schools by the change which he has initiated at Harvard.

I feel great sympathy with you, gentlemen, in having to listen to my remarks. I disclaim entirely all responsibility for them. You ought to have had a wiser man, a wittier speaker, a more profound thinker, to address you this evening, because it is a rare occasion. The whole onus of the fault of calling me up should rest upon the shoulders of Professor Brackett, and I ask you, therefore, to bestow your disapprobation upon him alone, and I further ask both him and you to accept my thanks for your great courtesy and your generous hospitality.

